# Optimizing Adaptive Disturbance Rejection Control Models Using the Chimp Optimization Algorithm for Ships' Hybrid Renewable Energy Systems

**DOI:** 10.1155/2022/3569261

**Published:** 2022-12-31

**Authors:** Ali Goudarzi Amlashi, Mohammad Rezvani, Mehdi Radmehr, Alireza Ghafouri

**Affiliations:** ^1^Department of Electrical Engineering, Azad University, Sari Branch, Sari, Iran; ^2^Department of Electrical Engineering, Azad University, Nour Branch, Nour, Iran

## Abstract

Hybrid renewable energy systems are becoming widely prevalent in warships due to their reliability and acceptability. However, the uncertainty caused by using renewable energy resources is one of the primary challenges. Therefore, this paper investigates the implementation of a dynamic voltage restorer (DVR) with a new control strategy in a hybrid solar power generation system, including photovoltaic (PV) panels, diesel generators, battery storage, and conventional and sensitive loads. Furthermore, a new metaheuristic-based active disturbance rejection control (ADRC) strategy for fast and accurate DVR control is proposed. In this regard, a novel chimp optimization algorithm (ChOA)-based (i.e., ChOA-ADRC) strategy is suggested to increase the stability and robustness of the aforementioned hybrid system. The ADRC controller's parameters are updated in real-time using the ChOA approach as an automatic tuning mechanism. In order to evaluate the performance of the proposed control strategy, the model is evaluated under two and three-phase fault case scenarios. Also, a comparison with the conventional PI controller has been performed to further evaluate the proposed method. Simulation findings reveal the suggested control strategy's remarkable effectiveness in correcting fault-caused voltage drop and maintaining sensitive load voltage. Additionally, the results show that ChOA-ADRC presents a better dynamic response compared to conventional control strategies and increases the reliability of the hybrid power generation system.

## 1. Introduction

In recent years, the reliability and availability of various power generation systems in ships have been widely investigated [1–3]. The greenhouse gases produced by ships increased [4]. Therefore, the international convention covering the prevention of pollution of the marine environment [5] has, in recent years taken measures aimed at reducing ships' greenhouse gas emissions [6, 7]. Integrating renewable energy sources is a solution to reduce environmental pollution and increase energy efficiency in conventional ship systems using only diesel generation-based systems [8, 9]. In this regard, a photovoltaic (PV)-based energy system has been used in several cases in recent years [10, 11].

Nevertheless, using a high amount of solar energy increases investment and reduces power system stability due to the uncertain nature of solar power [12–14]. Furthermore, numerous studies [15–21] showed that implementing an energy storage system (ESS) can be a very effective solution for increasing power system reliability and power quality, and it paves the way for increasing the penetration of renewable energy sources. Furthermore, the performance of electronic power switches has increased in capacitive storage, which has high reliability in heavy currents and high voltages [22, 23]. A ship power system equipped with PV alongside an ESS system can be considered a moving stand-alone microgrid.

In literature, several studies represented the power studies of hybrid power systems in ships [24, 25]. In [26], a battery system operation is investigated alongside a diesel generator for a ship crane. In [27], a battery system is studied to maximize fuel savings in electric power system-based ships. Several control strategies have been evaluated in [26] to increase battery life and reduce fuel consumption. A dynamic voltage restorer (DVR) eliminates a considerable portion of voltage sags and lowers the risk of load tripping and power loss during sags. Therefore, load stability can be guaranteed and reliability issues can be eliminated [28]. This paper uses a DVR for voltage compensation of sensitive loads in a hybrid solar energy generation system implemented on a ship and a control strategy to improve its dynamic performance.

Since the controller has always had trouble dealing with uncertainties and outside disturbances, sophisticated control techniques that can provide quick adaptivity and high robustness should be used. Numerous studies and applications of the disturbance/uncertainty estimation and attenuation (DUEA) methodologies have been conducted [29].

The ADRC, one of the DUEA approaches first introduced by Han [30] in 1995, has emerged as a reliable competitor to the traditional PID control approach. ADRC is a model-free error-based control technique. The two-degrees-freedom control architecture is extremely reliable and adaptable, but it can be challenging to fine-tune the controller's parameters, particularly in challenging environments like ships. It is necessary to tune and adjust the parameters of the ADRC controller in real-time in order to address the external disturbance and volatility concerns. Generally speaking, it has been a research area to figure out how to get a set of model parameters that can satisfy a particular performance index. A set of adjusted parameters can lead to improved control performance, which has significant implications for industrial manufacturing in terms of both economic consequences and environmental advantages. The performance and robustness of the control system can be improved with a properly designed and adjusted ADRC.

However, several parameters in the conceptual framework of the original ADRC need to be modified, and the computational complexity will rise as the proportional order of the control system rises. While there are many parameters, their interactions with one another add another level of complexity. Gao [31] suggested a bandwidth parameterization approach and LADRC with scaling. Although LADRC's parameter count is effectively down to two, the interaction effect between the parameters is still present and even gets more pronounced. Finding the ideal settings analytically in order to obtain optimum control performance is, therefore, fairly challenging. Additionally, when LADRC is used in industrial applications with time delays, the representative function of the system is a pseudopolynomial, making it much more difficult to find an analytical solution. Although it is simple to identify a collection of parameters that makes the control method stable, determining the best parameters for system performance has always been a challenge that necessitates quick research. Alternatively, the process of determining optimal control system settings can be viewed as an optimization model with the system performance metric as the fitness function [32]. The nonconvex nature of the optimization issue created by this method makes it challenging to solve using traditional optimization techniques, which motivates researchers to develop novel approaches to such problems.

Researchers have been focusing more and more on meta-heuristic algorithms to tackle engineering and practical challenges in recent years due to the advancement of digital computers. However, a few research studies apply metaheuristic algorithms for tuning the ADRC's parameters. For example, reference [33] used the particle swarm optimizer (PSO) to tune the ADRC's parameters. Although this approach was fast, it suffered from a low level of accuracy. Reference [34] utilized an adaptive differential ant-lion optimizer to optimize the parameter of ADRC. This approach presented high performance, but its main deficiency was high complexity. The multiobjective chimp optimization algorithm (ChOA) method is a promising way of fine-tuning the parameters since it uses less computation time to produce a stable and accurate solution. Our motivations for choosing ChOA [35] as an optimizer are as follows:

- No Free Lunch Theorem (NFL) [36]: This theorem states that no metaheuristic algorithm can solve all optimization problems. Testing is also the only way to find out the merits of a particular algorithm (in this case: ChOA) in solving a particular problem. Since the ChOA has not yet been applied to the ADRC's parameter tuning, we utilize this algorithm to solve the ADRC's parameter tuning problem for the first time.
- The merit of ChOA: ChOA has successfully improved and applied several optimization problems in various fields of study, including sonar dataset classification [37, 38], Covid19 diagnosis [39, 40], PV cell parameters identification [41], structure optimization [42], chemical engineering [43], industrial process optimization [44], binary and discrete optimization [45], node clustering and multihop routing protocol optimization [46], diagnosing Parkinson's disease [47], constraint multimodal engineering optimization [48], etc. The remainder of the paper is organized as follows: Section 2 presents the hybrid ship power system.  Section 3 represents the dynamic voltage restorer's concept. Afterward, Section 4 introduces the ADRC Control of DVR and the proposed ChOA-ADRC.  Section 5 represents the simulation results and discussions. Finally, the conclusion will be presented in Section 6.

## 2. Hybrid Ship Power System

In huge tanker ships, the propellers are powered by steam engines. The power system that supplies the electric loads of the ship is independent of these steam engines.

**Figure 1:**
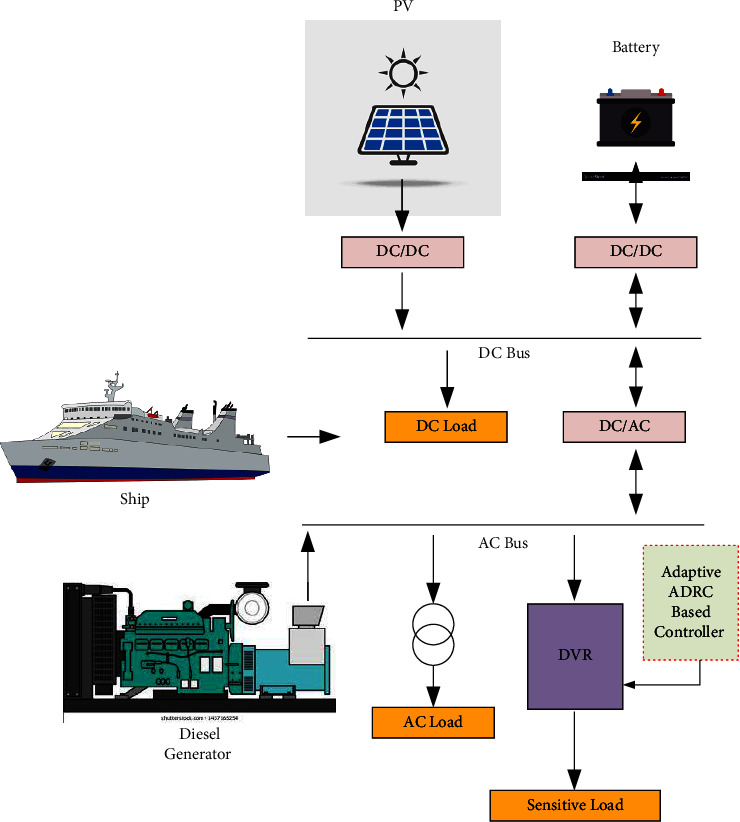
The ship hybrid solar energy generation system diagram.

The main difference between the land-isolated power system and the power system on the ship is the necessity of ensuring zero loss of load probability (LOLP) in the land-isolated power system. In contrast, the LOLP shall equal zero in a ship's power system. Therefore, implementing equipment to ensure a continuous power supply with high power quality is necessary for the ship's critical and sensitive loads. The ship's hybrid system consists of PV panels and their inverter, a diesel generator, and an ESS to store extra energy. As the power system in a ship operates as a stand-alone system all the time, the diesel generator rating must be at least equal to the sum of all loads together. In addition, DVR equipment with the proposed control strategy is implemented in the system to ensure a continuous supply of sensitive load under fault conditions.  Figure 1 illustrates the ship's hybrid solar energy generation system diagram.

## 3. Dynamic Voltage Restorer

**Figure 2:**
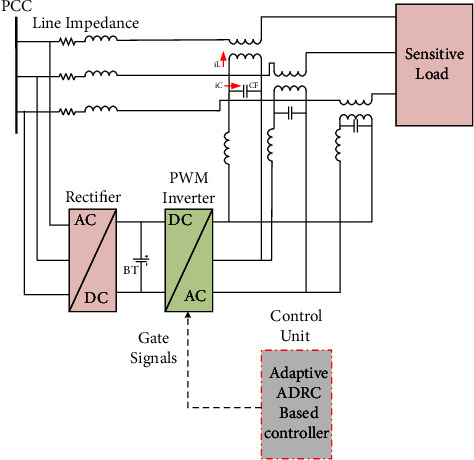
Dynamic voltage restorer structure with adaptive ADRC controller.

The DVR uses a voltage source inverter (VSI) which inverts the DC voltage of the capacitor dc-link into an AC voltage. Then, the voltage is injected into the system using three single-phase series transformers.  Figure 2 illustrates the dynamic voltage restorer structure with an adaptive ADRC controller.

The voltages and currents in the *d*-*q* synchronous reference frame can be expressed as

(1) $$\begin{array}{c} {\dot{i}}_{d}=\frac{1}{L_{f}}v_{d}+\omega i_{q}-\frac{1}{L_{f}}v_{cd}\text{,}\\ {\dot{i}}_{q}=\frac{1}{L_{f}}v_{q}-\omega i_{d}-\frac{1}{L_{f}}v_{cq}\text{,}\\ {\dot{v}}_{cd}=\frac{1}{C_{f}}i_{d}+\omega v_{cq}-\frac{1}{C_{f}}i_{ld}\text{,}\\ {\dot{v}}_{cq}=\frac{1}{C_{f}}i_{q}-\omega v_{cd}-\frac{1}{C_{f}}i_{lq}\text{,} \end{array}$$

where *v*_*dq*_ is the output voltage of the inverter on *d*-*q* axis, *v*_*cdq*_ is the filter capacitor on *d*-*q* axis, *i*_dq_ is the output current of the inverter on *d*-*q* axis, *I*_ldq_ is the load current on *d*-*q* axis, and *w* is the angular frequency of the source. The state-space model can be expressed as

(2) $$\begin{array}{c} \left\{\begin{array}{c} \dot{x}=Ax+Bu+Hd\text{,}\\ y=Cx\text{,} \end{array}\right. \end{array}$$

where *x* in the above equation is the state variables matrix, the matrix *u* is the input variable matrix, and *d* is the disturbance variable, and they all can be expressed as

(3) $$\begin{array}{c} \left\{\begin{array}{c} \dot{x}=Ax+Bu+Hd,\\ y=Cx, \end{array}\right.\\ u=\left[\begin{array}{c} u_{1}\\ u_{2} \end{array}\right]=\left[\begin{array}{c} v_{d}\\ v_{q} \end{array}\right]\text{,}\\ d=\left[\begin{array}{c} d_{1}\\ d_{2} \end{array}\right]=\left[\begin{array}{c} i_{ld}\\ i_{lq} \end{array}\right]\text{.} \end{array}$$

In DVR, the *A*, *B*, *C*, and *H* can be expressed as

(4) $$\begin{array}{c} A=\left[\begin{array}{cc} \begin{array}{cc} 0 & \omega \\ -\omega & 0 \end{array} & \begin{array}{cc} -\frac{1}{L_{f}} & 0\\ 0 & -\frac{1}{L_{f}} \end{array}\\ \begin{array}{cc} -\frac{1}{C_{f}} & 0\\ 0 & \frac{1}{C_{f}} \end{array} & \begin{array}{cc} 0 & \omega \\ -\omega & 0 \end{array} \end{array}\right]\text{,}\\ B=\left[\begin{array}{c} \begin{array}{c} \begin{array}{cc} \frac{1}{L_{f}} & 0 \end{array}\\ \begin{array}{cc} 0 & \frac{1}{L_{f}} \end{array} \end{array}\\ \begin{array}{c} \begin{array}{cc} 0 & 0 \end{array}\\ \begin{array}{cc} 0 & 0 \end{array} \end{array} \end{array}\right]\text{,}\\ \begin{array}{c} C=\left[\begin{array}{cc} \begin{array}{cc} 0 & 0\\ 0 & 0 \end{array} & \begin{array}{cc} 1 & 0\\ 0 & 1 \end{array} \end{array}\right],\\ H=\left[\begin{array}{c} \begin{array}{c} \begin{array}{cc} 0 & 0 \end{array}\\ \begin{array}{cc} 0 & 0 \end{array} \end{array}\\ \begin{array}{c} \begin{array}{cc} -\frac{1}{C_{f}} & 0 \end{array}\\ \begin{array}{cc} 0 & -\frac{1}{C_{f}} \end{array} \end{array} \end{array}\right]. \end{array} \end{array}$$

## 4. ADRC Control of DVR

The ADRC estimates and compensates for various external and internal disturbances in real-time. The control strategy is based on using an extended state observer (ESO). This paper develops the ChOA-ADRC to reduce the model complexity.

### 4.1. Adaptive Active Disturbance Rejection

Considering *u* (*t*) and *y* (*t*) as the input and output signals of a nonlinear process time variable with *m* dimensions, we can write the following equation:

(5) $$\begin{array}{c} y^{\left(m\right)}\left(t\right)=d_{\mathrm{int}}\left(y\left(t\right),y^{\left(1\right)}\left(t\right),y^{\left(2\right)}\left(t\right),\cdots ,y^{\left(m-1\right)}\left(t\right),u\left(t\right)\right)\\ +d_{ext}\left(t\right)+b_{0}u\left(t\right)\text{,} \end{array}$$

where i the *d*_int_(*y*(*t*), *y*^(1)^(*t*), *y*^(2)^(*t*), ⋯, *y*^(*m* − 1)^(*t*), *u*(*t*)) represents the nonlinear unknown dynamic of the system. The *d*_ext_(*t*) and *b*_0_ represent the external disturbances, respectively. These external disturbances are known system parameters.

Considering all affecting disturbances of the system can be controlled together as *d* (*t*) = *d*_int_ (*t*) + *d*_ext_ (*t*), the system can be presented by

(6) $$\begin{array}{c} y^{\left(m\right)}\left(t\right)=d\left(t\right)+b_{0}u\left(t\right)\text{.} \end{array}$$

The adaptive ADRC control strategy suggests that rather than finding a model of d (t), f (t) can be estimated and then canceled using a suitable control signal in real-time to reduce the control strategy's dependence on an accurate system modeling.

Thus, the canonical form of linear ADRC can be expressed as

(7) $$\begin{array}{c} \begin{array}{c} u=\left[\begin{array}{c} u_{1}\\ u_{2} \end{array}\right]\\ =\left[\begin{array}{c} v_{d}\\ v_{q} \end{array}\right], \end{array}\\ \begin{array}{c} y=\left[\begin{array}{c} y_{1}\\ y_{2} \end{array}\right]\\ =\left[\begin{array}{c} x_{3}\\ x_{4} \end{array}\right]\\ =\left[\begin{array}{c} v_{cd}\\ v_{cq} \end{array}\right]. \end{array} \end{array}$$

### 4.2. ESO Design

The utilized Luenberger observer (ESO) estimates f (t) and then compensates for the effect of disturbances that affect the system. Thus, a vector that includes states can be considered with an extended state that represents the system disturbances as a whole as

(8) $$\begin{array}{c} x={\left[x_{1}x_{2}\cdots x_{m-1}x_{m}x_{m+1}\right]}^{t}\\ ={\left[y\dot{y\cdots y_{m}d\left(t\right)}\right]}^{t}\text{.} \end{array}$$

The system can be represented as

(9) $$\begin{array}{c} \left\{\begin{array}{c} \dot{x}=Ax+Bu+E\dot{d}\text{,}\\ y=Cx\text{.} \end{array}\right. \end{array}$$

The ESO can be designed by

(10) $$\begin{array}{c} \left\{\begin{array}{c} \dot{x}=A\hat{x}+Bu+L\left(y-\hat{y}\right)\text{,}\\ \hat{y}=C\hat{x}\text{,} \end{array}\right. \end{array}$$

where the *L* is the gain of the observer model, and $\hat{x}$ is the predicted state-space variables, and for DVR, they can be expressed as

(11) $$\begin{array}{c} \begin{array}{c} \hat{x}=\left[\begin{array}{c} {\hat{x}}_{1}\\ {\hat{x}}_{2}\\ {\hat{x}}_{3}\\ {\hat{x}}_{4}\\ {\hat{x}}_{5}\\ {\hat{x}}_{6} \end{array}\right]\\ =\left[\begin{array}{c} {\hat{i}}_{d}\\ {\hat{i}}_{q}\\ {\hat{v}}_{cd}\\ {\hat{v}}_{cq}\\ {\hat{i}}_{ld}\\ {\hat{i}}_{lq} \end{array}\right], \end{array}\\ L=\left[\begin{array}{c} \begin{array}{cc} \beta_{11} & \beta_{12}\\ \beta_{21} & \beta_{22}\\ \beta_{31} & \beta_{32} \end{array}\\ \begin{array}{cc} \beta_{41} & \beta_{42}\\ \beta_{51} & \beta_{52}\\ \beta_{61} & \beta_{62} \end{array} \end{array}\right]\text{.} \end{array}$$

The difference between *x* and $\hat{x}$ can be expressed as

(12) $$\begin{array}{c} \varepsilon =x-\hat{x}\text{.} \end{array}$$

Thus, the error can be estimated as

(13) $$\begin{array}{c} \dot{\varepsilon }=\left(A-LC\right)\varepsilon \text{,} \end{array}$$

where

(14) $$\begin{array}{c} A-LC=\left[\begin{array}{c} \begin{array}{c} \begin{array}{cc} \begin{array}{ccc} 0 & \omega & -\frac{1}{L_{f}}-\beta_{11} \end{array} & \begin{array}{ccc} -\beta_{12} & 0 & 0 \end{array} \end{array}\\ \begin{array}{cc} \begin{array}{ccc} -\omega & 0 & -\beta_{21} \end{array} & \begin{array}{ccc} -\frac{1}{L_{f}}-\beta_{22} & 0 & 0 \end{array} \end{array}\\ \begin{array}{cc} \begin{array}{ccc} \frac{1}{C_{f}} & 0 & -\beta_{31} \end{array} & \begin{array}{ccc} \omega -\beta_{32} & -\frac{1}{C_{f}} & 0 \end{array} \end{array} \end{array}\\ \begin{array}{c} \begin{array}{cc} \begin{array}{ccc} 0 & \frac{1}{C_{f}} & -\omega -\beta_{41} \end{array} & \begin{array}{ccc} -\beta_{42} & 0 & -\frac{1}{C_{f}} \end{array} \end{array}\\ \begin{array}{cc} \begin{array}{ccc} 0 & 0 & -\beta_{51} \end{array} & \begin{array}{ccc} -\beta_{52} & 0 & 0 \end{array} \end{array}\\ \begin{array}{cc} \begin{array}{ccc} 0 & 0 & -\beta_{61} \end{array} & \begin{array}{ccc} -\beta_{62} & 0 & 0 \end{array} \end{array} \end{array} \end{array}\right]\text{.} \end{array}$$

To make sure of error estimation convergence, the gains vector *L* in the ESO should be chosen in a way that (*A* − LC) forms a Hurwitz matrix. It means that the poles of (*A* − LC) matrix polynomial characteristic (*P*ESO(*s*) of (*A* − LC)) must have negative real parts.

(15) $$\begin{array}{c} P_{ESO}\left(s\right)=\det\;\left(sI-\left(A-LC\right)\right)\text{.} \end{array}$$

**Figure 3:**
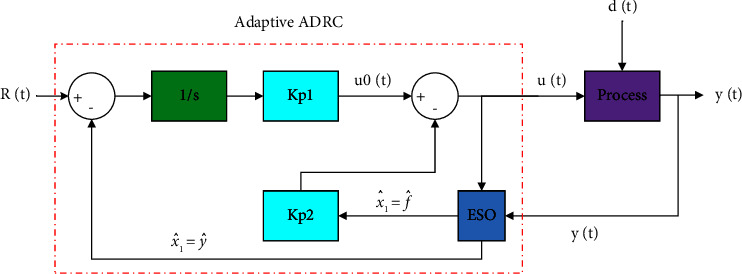
Adaptive ADRC-based control structure.

The necessary observer gains can be calculated for the location of the common pole through the polynomial characteristics.  Figure 3 illustrates the adaptive ADRC-based control strategy.

Figure 3 illustrates the adaptive ADRC-based control strategy.

### 4.3. Implementation of ChOA-ADRC

The ChOA approach is used to optimize the parameters so that the transient response meets the desired standards of least overshoot and minimum settling time. Each chimp in this method keeps track of the positions in the problem area that correspond to the optimum solution, which it has so far found.

When ChOA first begins, chimpanzees are generated at random. The chimpanzees are divided into four groups, each given a different mathematical model. It has been suggested that equation (16) can be used to describe a predator's urge to pursue and follow its prey [35]:

(16) $$\begin{array}{c} POS_{chimp}\left(k+1\right)=POS_{\text{prey}}\left(t\right)-b\cdot \left|d\cdot POS_{\text{prey}}\left(k\right)-ch\cdot POS_{chimp}\left(k\right)\right|\text{,}\\ b=2\cdot N\cdot r_{1}-N\text{,}\\ \begin{array}{c} d=2r_{2},\\ ch=Chaotic-vector. \end{array} \end{array}$$

Where *k* stands for the number of iterations, **POS**_prey_ is the best solution so far, **POS**_chimp_ is the best position for the chimpanzee, and **b**, **d**, and **ch** are the coefficients' vectors. Additionally, *r*_1_ and *r*_2_ are randomly chosen values in the range (0, 1], and **N** is a vector that is nonlinearly decreased from 2.5 to 0 during the iterations; **ch** is a vector created from a number of chaotic mappings. In the reference [35], these maps are described in detail.

Because it is unknown where the first prey was positioned in the environment, the best chimpanzees are utilized as prey to quantitatively reproduce chimp behavior. As a result, other agents will be forced to relocate in a manner proportional to the new locations of the four top chimpanzees, which ChOA will maintain, as defined by equations (17) and (18) [35].

(17) $$\begin{array}{c} POS\left(k+1\right)=\frac{1}{4}\times \left(POS_{1}+POS_{2}+POS_{3}+POS_{4}\right)\text{,} \end{array}$$

where

(18) $$\begin{array}{c} POS_{1}=POS_{Attacker}-b_{1}.\left|d_{1}POS_{Attacker}-ch_{1}POS\right|\text{,}\\ POS_{2}=POS_{Barrier}-b_{2}.\left|d_{2}POS_{Barrier}-ch_{2}POS\right|\text{,}\\ POS_{3}=POS_{Chaser}-b_{3}.\left|d_{3}POS_{Chaser}-ch_{3}POS\right|\text{,}\\ POS_{4}=POS_{Driver}-b_{4}.\left|d_{4}POS_{Driver}-ch_{4}POS\right|\text{.} \end{array}$$

Equation (19) shows that chaotic values can be used to imitate typical ChOA social incentive behavior.

(19) $$\begin{array}{c} POS_{chimp}\left(k+1\right)=\left\{\begin{array}{c} \begin{array}{cc} \text{Eq}.\left(5\right), & \lambda_{m}<0.5 \end{array}\text{,}\\ \begin{array}{cc} Chaotic-value, & \lambda_{m}\geq 0.5, \end{array} \end{array}\right. \end{array}$$

where *λ*_*m*_ stands for a random number between (0, 1]. The result of utilizing such a condensed model for learning could be an early or slow convergence rate.

#### 4.3.1. Fitness Function and Searching Agents

The operational integration of controller error *e* (*t*) is used as the fitness function in the majority of existing standards, including the Integral of Time multiplied by absolute error (ITAE) and integral of time multiplied by square error (ISTE), which have good technical application value. However, the controller parameters might be set rather high. Moreover, it takes a lot of work and effort to derive the mathematical equation. The system's overshoot and settling time amount are included as optimum objectives in the fitness function in order to enhance the system's dynamic behavior.

(20) $$\begin{array}{c} Fitness=\log\;\lambda +\log\;\varphi +\int_{0}^{\infty }e\left(t\right)dt\text{,} \end{array}$$

where the system's overshoot quantity and settling time, respectively, are *λ* and *φ*. The difference between the actual and desired values is expressed as *e* (*t*). This research describes the ChOA-ADRC controller, which uses the ChOA technique to find the ADRC controller's ideal controller parameters. The goal is to build a real-time configuration that uses ChOA to enhance ADRC. According to equation (11), each chimp has 12 individuals.

## 5. Simulation Results

In this part, the simulation results of implementing DVR with an adaptive ChOA-ADRC control strategy are presented and evaluated for application in the ship's hybrid solar energy generation system using MATLAB/Simulink 2021a software.

**Figure 4:**
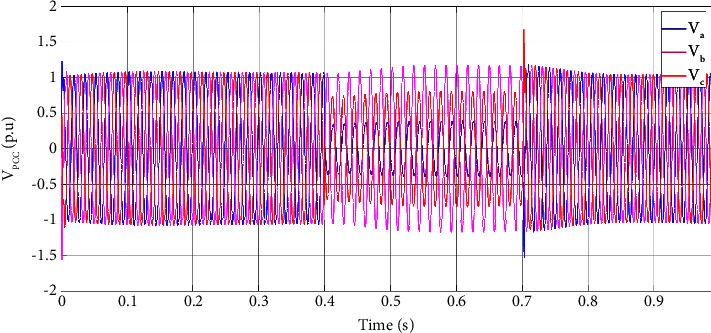
PCC voltage at (0, 1] time interval in a two-phase fault scenario.

A two-phase and a three-phase fault are simulated for a duration of 0.3 s to evaluate the dynamic performance of DVR with the suggested control strategy.  Figure 4 illustrates the PCC voltage when a two-phase fault accrues at 0.4 s and clears at 0.7 s. During the fault, the PCC voltage of the faulty phases drops. This dropped voltage below the acceptable voltage variation can damage the sensitive load and interrupt its normal operation.

To ensure the DVR's proper performance in case of a faulty occurrence, the minimum rating of the DVR should be equal to the load rating.

**Figure 5:**
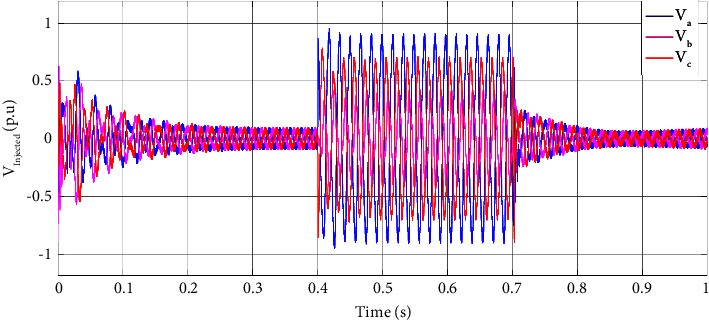
DVR injected voltage at (0, 1] time interval in a two-phase fault scenario.

The DVR with the proposed control strategy detects the voltage sag at the sensitive load's terminals by comparing the feedback voltage with the reference p.u. voltage. The controller produces a suitable reference voltage to be used by the inverter to compensate for the voltage sag. In Figure 5, the injected voltage by the DVR to compensate for the voltage sag of two faulty phases is illustrated at sensitive load terminals. The injected voltage of the DVR rises as the voltage drops to compensate for the voltage sag at 0.4 s and continues until the fault clears out.

**Figure 6:**
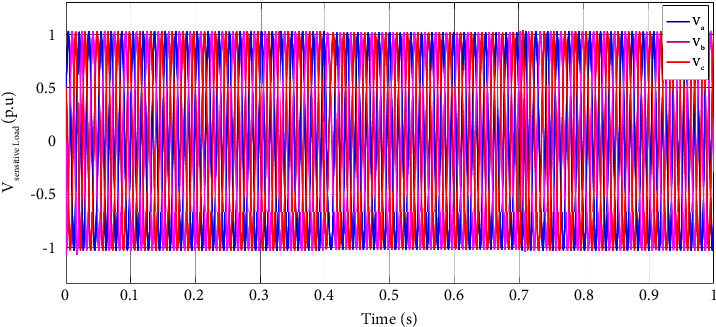
Sensitive load voltage at (0, 1] time interval in a two-phase fault scenario.

Figure 6 illustrates the sensitive load voltage as the hybrid generation system is under a two-phase fault from 0.4 s to 0.7 s. According to Figure 6, the voltage sag is clearly compensated completely, and the sensitive load voltage almost remains at p.u. during the fault.

**Figure 7:**
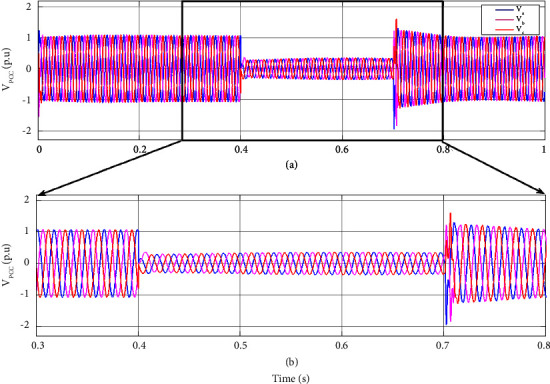
PCC voltage at (a) (0, 1] time interval and (b) (0.3, 1] time interval in three-phase fault.

For a three-phase fault case scenario, a three-phase fault is simulated for a duration of 0.3 s to evaluate the dynamic performance of the suggested control strategy.  Figure 7 illustrates the PCC voltage when a three-phase fault accrues at 0.4 s and clears at 0.7 s. During the fault, the PCC voltage drops to 0.03 p.u, approximately. This voltage is far below the acceptable voltage variation and can damage the sensitive load and interrupt its normal operation.

**Figure 8:**
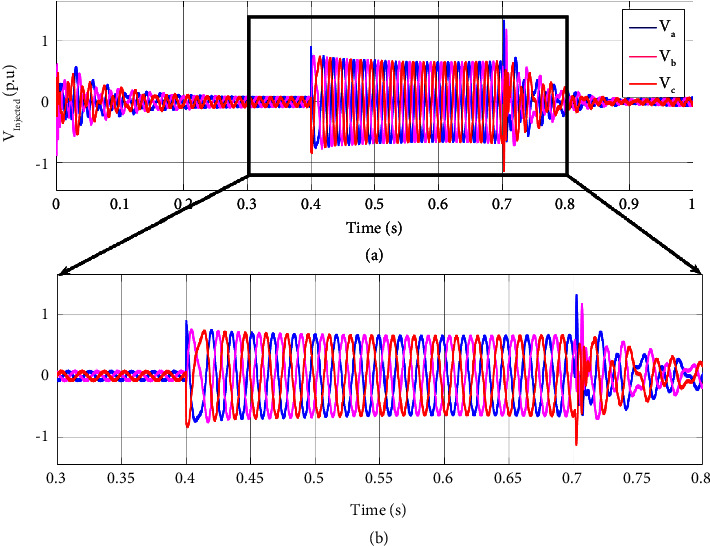
DVR injects voltage at (a) (0, 1] time interval and (b) (0.3, 1] time interval in a three-phase fault scenario.

Similar to the two-phase fault scenario, the injected voltage by DVR for a three-phase fault is illustrated in Figure 8 to compensate for the voltage sag of three faulty phases at sensitive load terminals. The injected voltage of DVR rises as the voltage drops to compensate for the voltage sag at 0.4 s and continues till the fault clears out.

**Figure 9:**
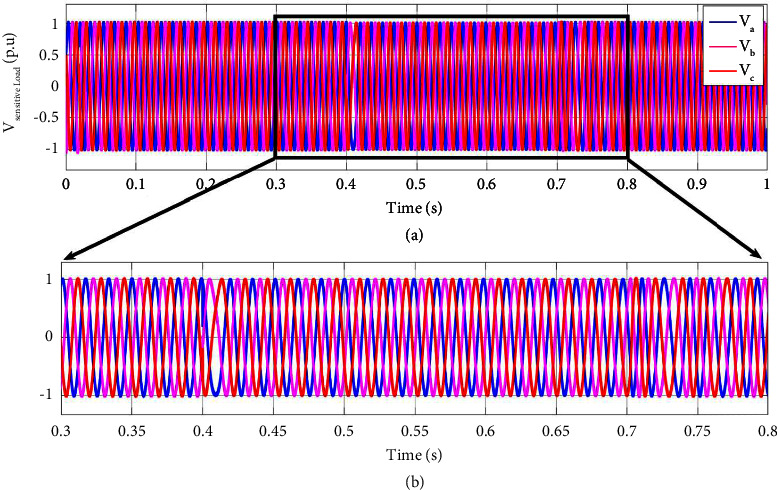
Sensitive load voltage at, (a) (0, 1] time interval, (b) (0.3, 1] time interval in a three-phase fault scenario.

Figure 9 illustrates the sensitive load voltage as the hybrid generation system is under a three-phase fault from 0.4 s to 0.7 s. According to Figure 6, the voltage sag is clearly compensated completely, and the sensitive load voltage almost remains one p.u. during the fault.

**Figure 10:**
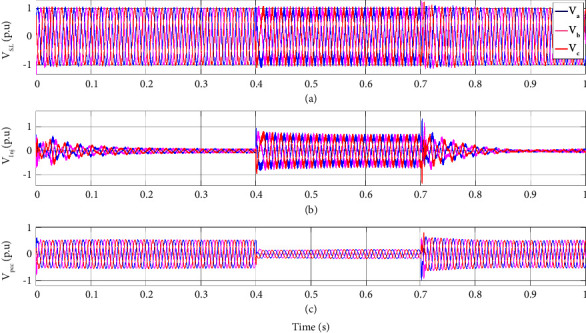
System voltages under fault. (a) sensitive load voltage, (b) injected voltage by DVR, and (c) PCC voltage in a three-phase fault scenario.

**Figure 11:**
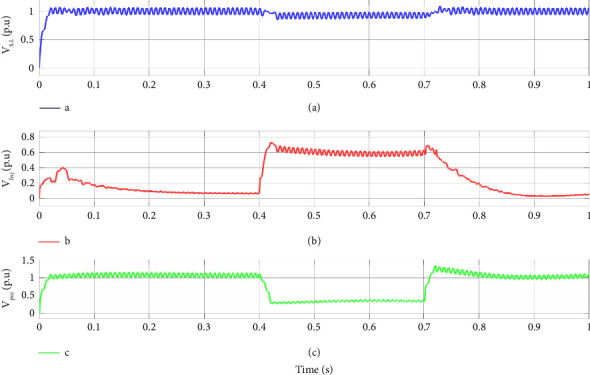
System r.m.s voltages under fault. (a) sensitive load voltage, (b) injected voltage by DVR, and (c) PCC voltage in three-phase fault scenario.

For a better illustration of DVR performance under system fault, the sensitive load voltage, the injected voltage by DVR, and the PCC voltage of the hybrid system under fault are illustrated for the 3 phase amplitude and r.m.s of the voltages in Figures 10 and 11, respectively. As the PCC voltage drops at 0.4 s, the DVR activates rapidly, and the adaptive ChOA-ADRC controller produces the proper reference voltage to be made by the DRV's inverter in order to compensate for the voltage sag. Thus, the voltage at sensitive load terminals remains almost intact, and no voltage drop is sensed. As the fault clears out, the injected voltage by DVR decreases to make a smooth transient for the sensitive load. This illustrates the effectiveness of the proposed control strategy of DVR for maintaining the sensitive load voltage under fault.

**Figure 12:**
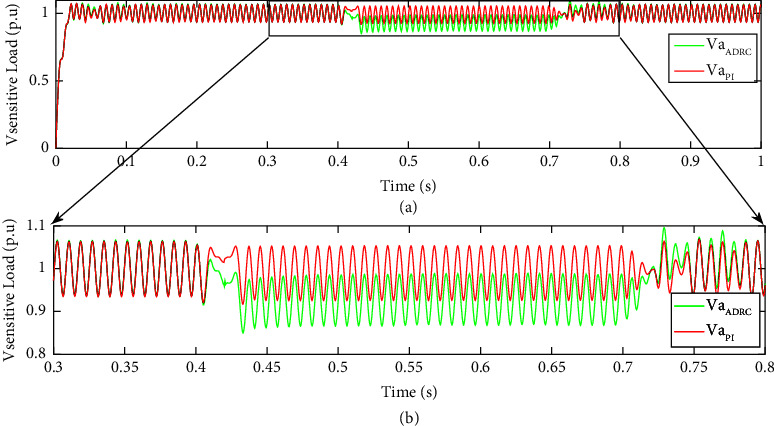
Dynamic performance comparison of the proposed ChOA-ADRC control strategy and PI-based controller. (a) (0.3, 0.9] time interval and (b) (0.3, 0.8] zoomed time interval in a three-phase fault scenario.

For more investigation of the proposed control strategy based on the novel ChOA-ADRC, a comparison is conducted with the conventional PI controller-based strategy.  Figure 12 shows the dynamic performance comparison of the proposed ChOA-ADRC control strategy and PI-based controller. The response time for the proposed ChOA-ADRC control strategy to reach steady-state after a fault is less than 0.05 s, and for the PI-based controller, it is 0.15 s. Clearly, the proposed control strategy presents a better dynamic response in terms of maintaining the sensitive load' r.m.s voltage at one p.u. Compared to conventional PI controllers.

## 6. Conclusion

This paper presented a novel control strategy based on ChOA-ADRC for fast and accurate control of DVR equipment in a war ship hybrid solar energy generation system, including PV panels, diesel generators, and normal and emergency loads. In order to evaluate the performance of the proposed control strategy, a simulation was carried out in MATLAB/Simulink 2021a software, and the system model was evaluated under fault conditions. The simulation results indicated the effectiveness of the proposed control strategy in compensating the voltage sag due to the fault in the system and maintaining the voltage of a sensitive load at one p.u. In addition, a comparison was performed for a more thorough evaluation of the proposed method with the conventional PI controller, and the results demonstrated a better dynamic response to the proposed control strategy. In terms of cost, the proposed controller is almost as expensive as the PI controller for inverters with programmable microcontrollers.

Furthermore, the results showed a better dynamic response to the proposed control strategy of the combined energy production system in the warship, which increased the reliability and availability of more equipment and sensitive loads in the warship and the defense factor.

Utilizing other novel metaheuristic algorithms, including the arithmetic optimization algorithm, marine predator algorithm, and salp swarm optimizer, can be considered one of the future research directions. Incorporating novel techniques such as the chaotic map, levy flight, and Nelder–Mead simplex approach into ChOA can be considered another research direction.

## Data Availability

Access to data is restricted.

## Additional Points

Simulation file is available. Models can be made available upon request.
